# Fabrication and Characteristics of a Three-Axis Accelerometer with Double L-Shaped Beams

**DOI:** 10.3390/s20061780

**Published:** 2020-03-24

**Authors:** Ying Wang, Xiaofeng Zhao, Dianzhong Wen

**Affiliations:** The Key Laboratory of Electronics Engineering, College of Heilongjiang Province, Heilongjiang University, Harbin 150080, China; 2181212@s.hlju.edu.cn (Y.W.); wendianzhong@hlju.edu.cn (D.W.)

**Keywords:** three-axis accelerometer, double L-shaped beam, MEMS technology, cross-axis sensitivity

## Abstract

A three-axis accelerometer with a double L-shaped beams structure was designed and fabricated in this paper, consisting of a supporting body, four double L-shaped beams and intermediate double beams connected to two mass blocks. When applying acceleration to the accelerometer chip, according to the output voltage changes of three Wheatstone bridges constituted by twelve piezoresistors on the roots of the beams, the corresponding acceleration along three axes can be measured based on the elastic force theory and piezoresistive effect. To improve the characteristics of the three-axis accelerometer, we simulated how the width of the intermediate double beams affected the characteristics. Through optimizing the structure size, six chips with different widths of intermediate double beams were fabricated on silicon-on-insulator (SOI) wafers using micro-electromechanical systems (MEMS) technology and were packaged on printed circuit boards (PCB) by using an electrostatic bonding process and inner lead bonding technology. At room temperature and *V*_DD_ = 5.0 V, the resulting accelerometer with an optimized size (*w* = 500 μm) realized sensitivities of 0.302 mV/g, 0.235 mV/g and 0.347 mV/g along three axes, with a low cross-axis sensitivity. This result provides a new strategy to further improve the characteristics of the three-axis accelerometer.

## 1. Introduction

In recent years, with the rapid development of semiconductor technology, accelerometers have been widely used in many different fields, mainly including vibration detection [1], consumer electronics [2], medical treatment [3,4], vibration detection of wind turbines [5], navigation [6,7], automobile safety [8], and so on. Currently, although accelerometers have met the needs of real life, they are still facing the challenge of a wide measurement range, high accuracy, etc. Thus, high performance accelerometers have stimulated a particular attention from researchers, providing an opportunity to improve the characteristics of the accelerometer, making it possible to overcome the above mentioned limitation. For example, Sankar et al. proposed a quad beam silicon piezoresistive *z*-axis accelerometer with a very low cross-axis sensitivity of 0.316 μV/V∙g for an in-plane acceleration in 2013 [9]. After that, Han et al. presented a piezoresistive accelerometer with a low cross-axis sensitivity based on masked-maskless wet etching, realizing sensitivities of 0.069 V/V∙g, 0.034 V/V∙g and 4.15 V/V∙g along the *x*-axis, *y*-axis and *z*-axis, and cross-axis sensitivities of 1.67% and 0.82% along the *x*-axis and *y*-axis, respectively [10]. Recently, Takahashi et al. reported an angular acceleration sensor composed of liquid ring channels and piezoresistive cantilevers as the sensing element, achieving an angular acceleration resolution of 0.01 rad/s^2^ in the range of 0.1–100 Hz on a target axis about 100 times higher than that of the other axes, a high sensitivity and a low cross-interference [11]. Nowadays, the accelerometer can be divided into several types, mainly including piezoresistive, capacitive [12], piezoelectric [13], etc. The piezoresistive accelerometer, when compared with the ones having other structures, exhibits many excellent properties, such as a fast response, easy signal processing and miniaturization, etc. However, the piezoresistive accelerometers are also facing challenges with respect to improving measurement consistency and reducing cross-interference, which have limited their further development.

To overcome the above obstacles, a piezoresistive three-axis accelerometer with double L-shaped beams was designed and fabricated on a silicon-on-insulator (SOI) wafer using MEMS technology in this study. Using the double L-shaped beams concentrated the stress distribution under the actions of *a_x_* and *a_y_*. Through a simulation to investigate how the width of the intermediate double beams affects the characteristics of an accelerometer, the structure size of the intermediate double beams was optimized. The test results indicate that it is possible to achieve the measurement of acceleration along three axes and reduce the cross-axis sensitivity using the resulting accelerometer. The study on the effects of intermediate double beams’ structure sizes on the sensitivity of the proposed accelerometer provides a possibility to further improve the performance of three-axis accelerometers. 

## 2. Basic Structure and Working Principle 

### 2.1. Basic Structure

**Figure 1:**
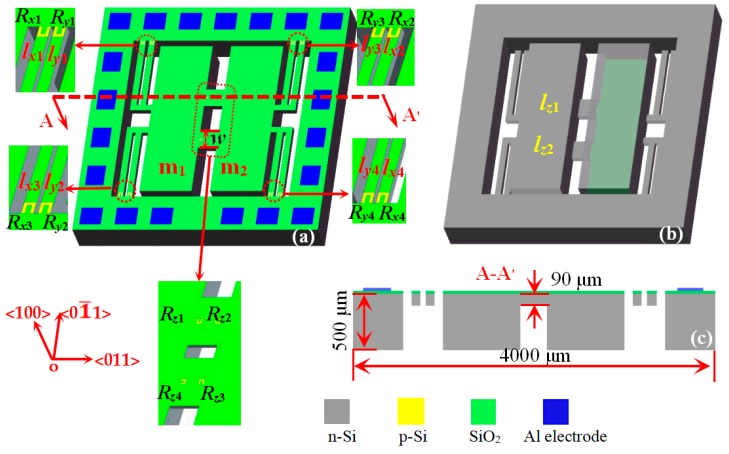
The basic structure of the three-axis accelerometer with double L-shaped beams: (**a**) top view; (**b**) bottom view; and (**c**) cross-section view along AA’.

The three-axis accelerometer is composed of an elastic element and a sensitive element, where the elastic element consists of four double L-shaped beams, intermediate double beams and two mass blocks, and the sensitive element has twelve piezoresistors. The basic structure of the three-axis accelerometer with double L-shaped beams is shown in Figure 1, including a top view, bottom views, as well as a cross-section view along AA’. As shown in Figure 1a,b, the two mass blocks (m_1_ and m_2_) are first connected by the intermediate double beams (l*_z_*_1_ and l*_z_*_2_) and are then connected to the support body by four double L-shaped beams (l_*x*1_, l_*x*2_, l_*x*3_, l_*x*4_, l_*y*1_, l_*y*2_, l_*y*3_ and l_*y*4_,) on a SOI wafer. Figure 1c gives the cross-section view along AA’ for the three-axis accelerometer, in which the thicknesses of the intermediate double beams are the same as those of the four double L-shaped beams.

As seen in Figure 1, the twelve piezoresistors (*R_x_*_1_, *R_x_*_2_, *R_x_*_3_, *R_x_*_4_, *R_y_*_1_, *R_y_*_2_, *R_y_*_3_, *R_y_*_4_, *R_z_*_1_, *R_z_*_2_, *R_z_*_3_ and *R_z_*_4_) were designed on the roots of four double L-shaped beams and intermediate double beams, where the four piezoresistors (*R_x_*_1_, *R_x_*_2_, *R_x_*_3_ and *R_x_*_4_) along the <$0\bar{1}1$ > orientation are fabricated on the four L-shaped single beams (l_*x*1_, l_*x*2_, l_*x*3_ and l_*x*4_) to form a Wheatstone bridge (W_1_). Meanwhile, the four piezoresistors (*R_y_*_1_, *R_y_*_2_, *R_y_*_3_ and *R_y_*_4_) along the <$0\bar{1}1$> orientation are fabricated on the four L-shaped single beams (l_*y*1_, l_*y*2_, l_*y*3_ and l_*y*4_) to constitute a Wheatstone bridge (W_2_), and the other piezoresistors (*R_z_*_1_, *R_z_*_2_, *R_z_*_3_ and *R_z_*_4_) along the <$0\bar{1}1$> and <011> orientations are respectively fabricated on the intermediate double beams (l*_z_*_1_ and l*_z_*_2_) to form a Wheatstone bridge (W_3_). In order to study the effect of the width (*w*) of the intermediate double beams (l*_z_*_1_ and l*_z_*_2_) on the characteristics of the proposed accelerometer, six chip sizes combined with the simulation in Section 3 of this study are shown in Table 1. 

### 2.2. Sensitivity Analysis

**Figure 2:**
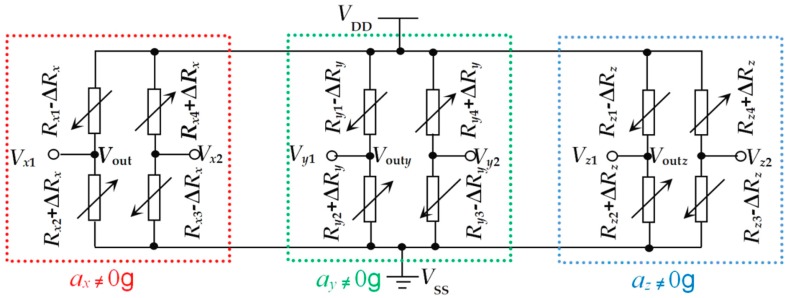
The equivalent circuit of the three-axis accelerometer under the action of *a_x_*, *a_y_* and *a_z_*.

Figure 2 shows the equivalent circuit of the proposed three-axis accelerometer under the actions of *a_x_*, *a_y_* and *a_z_*. When the supply voltage is *V*_DD_ and no acceleration is applied to the chip, the output voltages of three Wheatstone bridges consisting of twelve piezoresistors are zero due to the fact that the resistances of the twelve piezoresistors are the same under ideal conditions. When exerting acceleration *a_x_* along the *x*-axis to the chip, the four double L-shaped beams will produce deformations. According to the piezoresistive effect of the semiconductor material, the four piezoresistors of *R_x_*_1_, *R_x_*_2_, *R_x_*_3_ and *R_x_*_4_ on the roots of l_*x*1_, l_*x*2_, l_*x*3_ and l_*x*4_ are changed, i.e., the reducing of *R_x_*_1_ and *R_x_*_3_ attributed to the inducted compressive stress acted on the roots of l_*x*1_ and l_*x*3_, while in contrast the increases of *R_x_*_2_ and *R_x_*_4_ caused by the tensile stress acted on the roots of l_*x*2_ and l_*x*4_. The absolute variations of four piezoresistors are equal in ideal conditions, named as Δ*R_x_*. Due to the fact that the output voltages *V_x_*_1_ and *V_x_*_2_ of W_1_ are relative to the acceleration along the *x*-axis, it is possible to realize the measurement of *a_x_*. When applying the acceleration *a_y_* along the *y*-axis to the chip, the four double L-shaped beams will show deformations, and *R_y_*_1_, *R_y_*_2_, *R_y_*_3_ and *R_y_*_4_ located on the roots of l_*y*1_, l_*y*2_, l_*y*3_ and l_*y*4_ are changed, resulting in the reduction of *R_y_*_1_ and *R_y_*_3_ due to the induced compressive stress action on the roots of l_*y*1_ and l_*y*3_, as well as in the increase of *R_x_*_2_ and *R_x_*_4_ caused by the tensile stress acting on the roots of l_*y*2_ and l_*y*4_. The same absolute variations of four piezoresistors in ideal conditions are regarded as Δ*R_y_*. The change of the output voltages *V_y_*_1_ and *V_y_*_2_ of W_2_ under the action of *a_y_* makes it possible to achieve the measurement of *a_y_*. In addition, when exerting the acceleration *a_z_* along the *z*-axis to the chip, the intermediate double beams l*_z_*_1_ and l*_z_*_2_ will produce a deformation along the *z*-axis, and *R_z_*_1_, *R_z_*_2_, *R_z_*_3_ and *R_z_*_4_ on the roots of l*_z_*_1_ and l*_z_*_2_ are changed, indicating that *R_z_*_1_ and *R_z_*_3_ are influenced by compressive stress, leading to the decreases of *R_z_*_1_ and *R_z_*_3_ and the increases of *R_z_*_2_ and *R_z_*_4_. Similarly, the same absolute variations of four piezoresistors in ideal conditions are regarded as Δ*R_z_*. The changes of the output voltages *V_z_*_1_ and *V_z_*_2_ of W_3_ under the action of *a_z_* contribute to the measurement of *a_z_*.

When applying acceleration to the chip, the deformations of the proposed accelerometer beams would cause the variations of piezoresistors, where ∆*R_x_*, ∆*R_y_*, ∆*R*_z_ are the changes of piezoresistors under the actions of *a_x_*, *a_y_* and *a_z_*, respectively.

In an ideal case, *R_x_*_1_ = *R_x_*_2_ = *R_x_*_3_ = *R_x_*_4_ = *R_x_*_0_, *R*_*y*1_ = *R_y_*_2_ = *R_y_*_3_ = *R_y_*_4_ = *R_y_*_0_, *R_z_*_1_ = *R_z_*_2_ = *R_z_*_3_ = *R_z_*_4_ = *R_z_*_0_. In view of the equivalent circuit and piezoresistive effect, the relationship between the output voltages and the relative changes of piezoresistors can be expressed as follows [14]:

(1) $$\left\{ \begin{array}{l} V_{\mathrm{out}x}=V_{x1}-V_{x2}=\frac{\Delta R_{x}}{R_{x0}}\cdot V_{\mathrm{DD}}\\ V_{\mathrm{out}y}=V_{y1}-V_{y2}=\frac{\Delta R_{y}}{R_{y0}}\cdot V_{\mathrm{DD}}\\ V_{\mathrm{out}z}=V_{z1}-V_{z2}=\frac{\Delta R_{z}}{R_{z0}}\cdot V_{\mathrm{DD}} \end{array}\right.$$

where *R_x_*_0_, *R_y_*_0_ and *R_z_*_0_ are the piezoresistors under no acceleration, *V*_out*x*_, *V*_out*y*_ and *V*_out*z*_ are the output voltages of W_1_, W_2_, and W_3_, with the value of zero under the same condition.

In view of Equation (1), the output voltages of the Wheatstone bridges are directly proportional to the relative changes of the piezoresistors at a constant supply voltage. Thus, it is possible to measure the external acceleration based on the output voltage, where Δ*R* relative to stress is given in Equation (2) [15]:

(2) $$\frac{\Delta R}{R}=\pi_{l}\sigma_{l}+\pi_{t}\sigma_{t}$$

where *π_l_* and *π_t_* are the longitudinal and transverse piezoresistance coefficients, and *σ_l_* and *σ_t_* are the longitudinal and transverse stress of the piezoresistors, respectively.

Due to the fact that the proposed accelerometer was designed on the SOI wafer with a device layer of n-type silicon with a <100> orientation, through combining Equation (1) with Equation (2), the relationship between the output voltages and the stress can be expressed as Equation (3):

(3) $$\left\{ \begin{array}{l} V_{\mathrm{out}x}=\frac{V_{\mathrm{DD}}}{2}\cdot \pi_{44}(\sigma_{lx}-\sigma_{tx})\\ V_{\mathrm{out}y}=\frac{V_{\mathrm{DD}}}{2}\cdot \pi_{44}(\sigma_{ly}-\sigma_{ty})\\ V_{\mathrm{out}z}=\frac{V_{\mathrm{DD}}}{2}\cdot \pi_{44}(\sigma_{lz}-\sigma_{tz}) \end{array}\right.$$

where σ*_lx_* and σ_t*x*_ are the longitudinal and transverse stress of *R_x_*_1_, *R_x_*_2_, *R_x_*_3_ and *R_x_*_4_, σ*_ly_* and σ_t*y*_ are the longitudinal and transverse stress of *R_y_*_1_, *R_y_*_2_, *R_y_*_3_ and *R_y_*_4_, σ*_lz_* and σ_t*z*_ are the longitudinal and transverse stress of *R_z_*_1_, *R_z_*_2_, *R_z_*_3_ and *R_z_*_4_, *π*_44_ is piezoresistive coefficient.

According to the sensitivity definition and sensitive principle of the three-axis accelerometer, the output voltages of the Wheatstone bridges along three axes can be expressed as Equation (4):

(4) $$\left[\begin{array}{l} V_{\mathrm{out}x}\\ V_{\mathrm{out}y}\\ V_{\mathrm{out}z} \end{array}\right]=\left[\begin{array}{l} S_{xx}S_{xy}S_{xz}\\ S_{yx}S_{yy}S_{yz}\\ S_{zx}S_{zy}S_{zz} \end{array}\right]\left[\begin{array}{l} a_{x}\\ a_{y}\\ a_{z} \end{array}\right]$$

where *S_xx_*, *S_yy_* and *S_zz_* are the sensitivities along the *x*-axis, *y*-axis and *z*-axis, respectively. *S_xy_* and *S_xz_* are the cross-axis sensitivities along the *x*-axis under *a_y_* and *a_z_*, respectively. *S_yx_* and *S_yz_* are the cross-axis sensitivities along the *y*-axis under *a_x_* and *a_z_*, respectively. *S_zx_* and *S_zy_* are the cross-axis sensitivities along the *z*-axis under *a_x_* and *a_y_*, respectively. 

In an ideal case, the elastic deformations of the four double L-shaped beams under the action of *a_z_* are identical, resulting in the same absolute variations of piezoresistors on the four double L-shaped beams, without the changes of the output voltages of W_1_ and W_2_ as well as the changes of the cross-axis sensitivities (*S_xz_* and *S_yz_*) of the sensor. Similarly, all the cross-axis sensitivities of *S_yx_*, *S_zx_*, *S_zy_* and *S_xy_* are zero and can be ignored. Thus, Equation (4) can be simplified as Equation (5):

(5) $$\left[\begin{array}{l} V_{\mathrm{out}x}\\ V_{\mathrm{out}y}\\ V_{\mathrm{out}z} \end{array}\right]=\left[\begin{array}{lll} S_{xx} & 0 & 0\\ 0 & S_{yy} & 0\\ 0 & 0 & S_{zz} \end{array}\right]\left[\begin{array}{l} a_{x}\\ a_{y}\\ a_{z} \end{array}\right]$$

Based on the above theoretical analysis, it is possible to realize the measurement of acceleration along the *x*-axis, *y*-axis and *z*-axis by the accelerometer. Meanwhile, the cross-axis sensitivity can be ignored by structural optimization under the ideal process conditions and the ideal environmental testing conditions, etc.

## 3. Simulation and Fabrication Technology

### 3.1. Simulation of Three-axis Accelerometer

**Figure 3:**
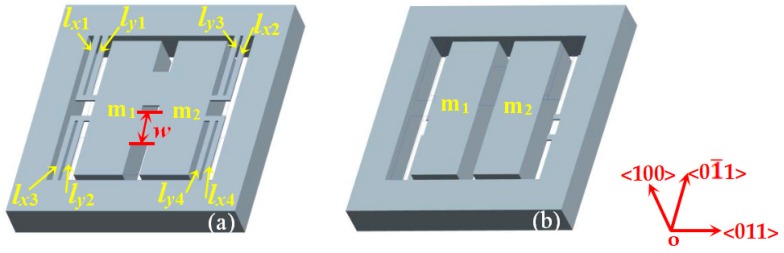
The structure model of the three-axis accelerometer: (**a**) top view; (**b**) bottom view.

To study how ***w*** affects the stress distribution, the finite element simulation software-Ansys 15.0 was used to simulate the characteristics of the proposed sensor, as shown in Figure 3. Figure 3a,b shows the top and bottom views of the sensor model with four double L-shaped beams, intermediate double beams and two mass blocks. 

**Figure 4:**
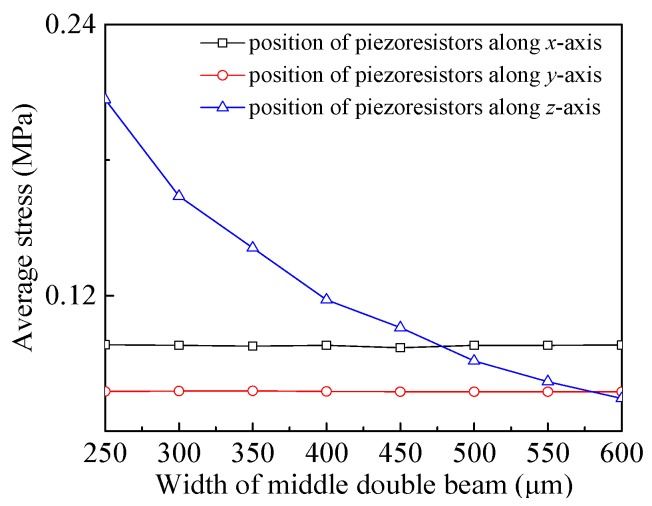
The relationship curves between the average stress at the piezoresistor positions along three axes and the width (*w*) of the intermediate double beams.

On the basis of the model, the relationship between the average stress at the piezoresistor positions on the beams and *w* under the actions of *a_x_*, *a_y_* and *a_z_* was investigated. By respectively exerting an external acceleration of 15 g along the *x*-axis, *y*-axis and *z*-axis to the sensor model, six sensors (see Table 1) with different *w* values were simulated. Then, the average stress at the corresponding piezoresistor positions was given, as shown in Figure 4. It can be seen that the average stress at the piezoresistor positions along teh *z*-axis decreases with the increase of *w*. With respect to that, the ones along the *x*-axis and *y*-axis do not change much, as shown in Figure 4. However, when increasing *w* up to 500 μm, the average stress differences along the three axes are very small, and the measurement consistency is good. According to the derived equation in Section 2, it can be found that *S_xx_*, *S_yy_* and *S_zz_* increase with the increase of the average stress, resulting in the decrease of the sensitivity along the *z*-axis with an increasing *w*. Finally, a better consistence of sensitivity is achieved when *w* is 500 μm.

### 3.2. The Fabrication Process of Three-axis Accelerometer 

**Figure 5:**
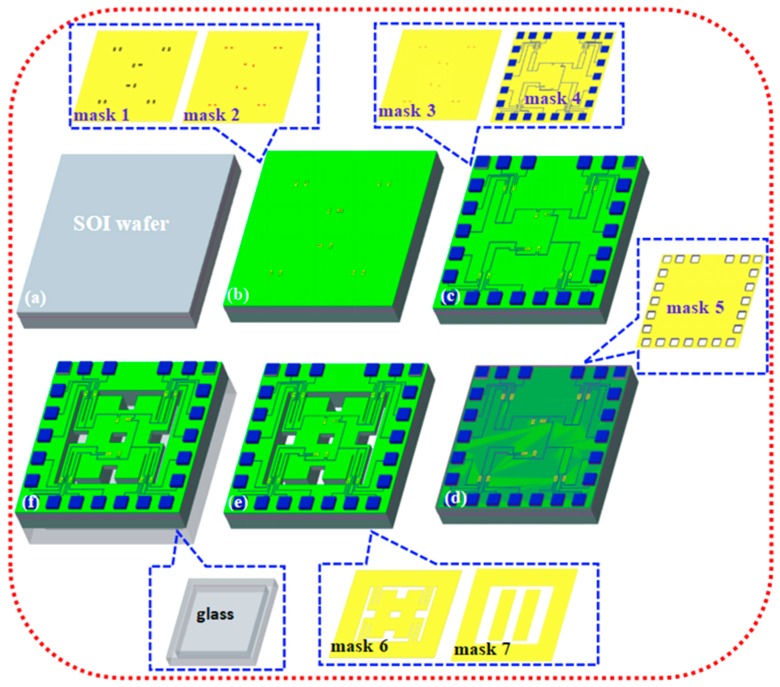
The main fabrication process of the proposed sensor (insets of mask1-mask7 used for photolithography and bonding glass): (**a**) cleaning the wafer; (**b**) forming p+ and p- regions; (**c**) performing contact holes to fabricate the electrodes and interconnects; (**d**) forming pad; (**e**) ICP etching the top and bottom surfaces of the chip to release the beam structure; and (**f**) bonding a glass plate with the bottom surface of chip.

As shown in Figure 5, the chip was fabricated on a SOI wafer with a n-type <100> orientation device layer and resistivity of 0.1 Ω·cm by using MEMS technology, in which the insets of mask1-mask7 used for photolithography and bonding glass are given together. The synthesis procedure is illustrated as follows:

(a) Cleaning a SOI wafer using a standard cleaning method, then growing a SiO_2_ layer with a thickness of 50 nm by thermal oxidation method as an ion implantation buffer; (b) first, using photolithography to form an ion implantation window, performing the p+ region by utilizing ion implantation, and secondly using photolithography to etch the windows of piezoresistors, performing the p- region as piezoresistors repeating the above method. After that, placing the chip in a vacuum environment of 1000 °C for 20~30 min to activate impurity ions so as to form an impurity distribution and effectively eliminate the damage caused by the ion implantation. Etching an SiO_2_ layer of 50 nm using wet etching technology, and thereafter growing the SiO_2_ layer as an insulating layer by using plasma enhanced chemical vapor deposition (PECVD); (c) third, photolithography to etch the top surface and performing contact holes between the piezoresistors and the Al electrodes, fabricating metal Al on the top surface by a vacuum evaporation method, and fourth, photolithography to form the electrodes and interconnects, and then metalizing at 420 °C for 30 min to achieve ohmic contact; (d) growing a SiO_2_ layer by using PECVD as a passive layer to protect the Al electrode, and fifth, photolithography to form a pad; (e) etching the bottom surface of the chip by inductive couple plasma (ICP) technology [16] to form two mass blocks, thereafter etching the top surface of the chip by the identical technology to release four double L-shaped beams and intermediate double beams; (f) bonding a glass plate with a groove with the bottom surface of the chip by using electrostatic bonding technology to provide enough moving space for the two mass blocks.

**Figure 6:**
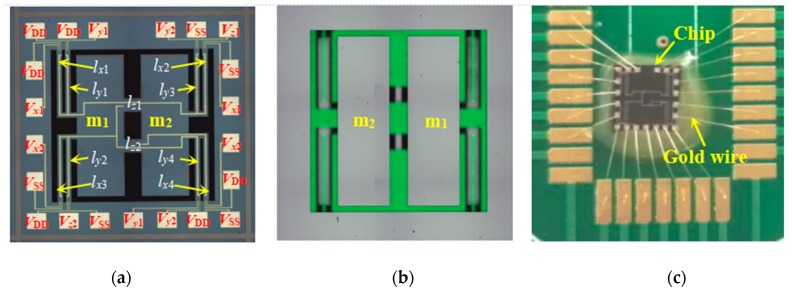
The photograph of the proposed chip: (**a**) top view; (**b**) bottom view; and (**c**) packaged chip.

To observe the morphology of the chip, a high-precision measuring microscope (Japan, Olympus, STM7) was used. Due to the large size (4000 μm×4000 μm) of the chip, the morphology stitching mode was used to complete the whole photograph. Figure 6a,b shows the top and bottom photographs of the fabricated chip, respectively. As shown in Figure 6a, the potential labeling of pad points on the chip corresponds to that in the equivalent circuit in Figure 2. Meanwhile, using the Dow Corning fluorosilicone solvent-resistant sealant attached the chip to the package-printed circuit board (PCB) [17]. After fully curing the sealant, bonding the leads between the chip and PCB using an integrated chip bonding machine (American, KNS, 4526) realized the connection between the solder joints on the chip and the corresponding solder joints on the PCB, as shown in Figure 6c [14].

## 4. Results and Discussion

To study the characteristics of the three-axis accelerometer, a testing system was built, consisting of a standard vibration table (China, Dongling, ESS-050) with an excitation frequency range of 0~10,000 Hz and acceleration of 0~30 g with an accuracy of 0.1 g, a high performance digital multimeter (America, Agilent, 34410A), and a programmable linear direct-current power (China, Rigol, DP832A) to supply a voltage of 0~30 V. The test was carried out at room temperature and a humidity of 40% RH.

### 4.1. Frequency Response Characteristics

**Figure 7:**
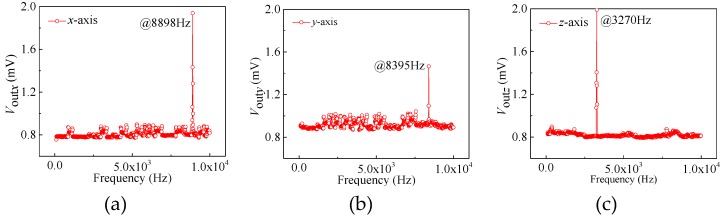
The characteristic curves of resonance frequency when *w* is 500 μm: (**a**) along the *x*-axis; (**b**) along the *y*-axis; and (**c**) along the *z*-axis.

To investigate the frequency response characteristics of the six types of sensors, a frequency response characteristic test was carried out under conditions of the supply voltage of 5.0 V and an external acceleration of 3 g with a frequency range from 100 Hz to 10,000 Hz. Through taking the chip with *w* = 500 μm as an example, the characteristic curves of the resonance frequency along the *x*-axis, *y*-axis and *z*-axis are respectively shown in Figure 7a–c. As shown in Figure 7a, the test result shows that the output voltages of the sensor do not markedly change with an increase of the excitation frequency. When increasing the frequency of the excitation signal up to 8898 Hz along the *x*-axis, the output voltage of the sensor along the *x*-axis reaches a maximum. Then, the output voltage of the sensor begins to gradually decrease as the vibration frequency continually increases, thus achieving the resonance frequency of 8898 Hz along the *x*-axis when *w* = 500 μm. Similarly, when *w* is 500 μm the resonance frequencies along the *y*-axis and *z*-axis are 8395 Hz and 3270 Hz, as shown in Figure 7b,c. After that, a wide frequency sweep was performed on the proposed sensor, i.e., with a vibration frequency range from 100 Hz to 10,000 Hz at constant acceleration. The resonance frequencies of the six types of chips (named as AS-1~AS-6, corresponding to different *w* values of 250–500 μm) were obtained by observing the output voltage changes, as shown in Table 2. According to Table 2, due to the fact that the two identical mass blocks in the basic structure of the accelerometer were connected by the intermediate double beams, a collective movement can be caused to form the deformations of the double L-shaped beams, benefitting the measurement of *a_x_* or *a_y_*. However, the widths of the intermediate double beams (*w*) have little influence on the movement along the *x*-axis or *y*-axis, without monotonous changes of the resonance frequency along the *x*-axis and *y*-axis with *w*.

### 4.2. Sensitivity and Cross Interference Characteristics

**Figure 8:**
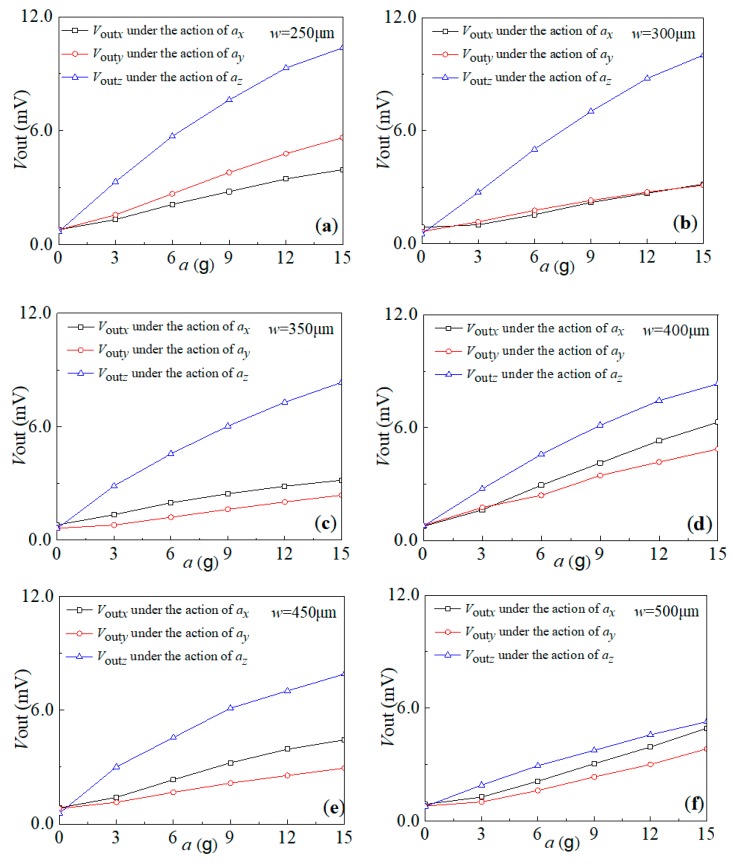
The relationship curves between the output voltages of the proposed sensor and the external acceleration of the six types of sensors with different *w* values: (**a**) *w* = 250 μm; (**b**) *w* = 300 μm; (**c**) *w* = 350 μm; (**d**) *w* = 400 μm; (**e**) *w* = 450 μm; and (**f**) *w* = 500 μm.

To research the sensitivity characteristics of the six types of sensors, the sensitivity test was repeated three times under the conditions of a supply voltage of 5.0 V and acceleration along the sensitive axis from 0 g to 15 g with a step of 3 g. Figure 8 gives the relationship curves between the output voltages and the external acceleration along the three axes, where *V*_out*x*_ (see the black line), *V*_out*y*_ (see the red line) and *V*_out*z*_ (see the blue line) are the output voltages for the resulting sensors. As shown in Figure 8a, it can be seen that the output voltages *V*_out*x*_ and *V*_out*y*_ of the AS-1 sensor linearly increase with an increase of *a_x_* and *a_y_*, respectively. With respect to that, the output voltage *V*_out*z*_ of the sensor nonlinearly increases with an increase of *a_z_*. In addition, when *a_x_* = *a_y_* = *a_z_*, *V*_out*z*_ are markedly bigger than *V*_out*x*_ and *V*_out*y*_, where *V*_out*y*_ is close to *V*_out*x*_. This indicates that the resulting AS-1 sensor can realize the acceleration measurement along the three axes, but with a low linearity and higher sensitivity along the *z*-axis when compared with the other axes. As shown in Figure 8b–e, the sensors of AS-2, AS-3, AS-4 and AS-5, when compared with AS-1, have similar sensitivities and linearity along the three axes. In particular, the corresponding output voltages of the AS-6 sensor linearly increase, not only with the increasing of the external accelerations *a_x_* and *a_y_*, but also with the increasing of *a_z_*, as shown in Figure 8f. In addition, when *a_x_* = *a_y_* = *a_z_*, *V*_out*x*_, *V*_out*y*_ and *V*_out*z*_ are close, all having a relationship curve with an approximate linearity. On the basis of this, the characteristics of the AS-6 sensor are in accord with the previous simulating results.

In addition, according to the sensitivity definition and testing data, the sensitivities of the proposed sensor with different *w* values are shown in Table 3. It indicates that the *sensitivities* of three-axis accelerometers decrease along the *z*-axis with the increasing widths of the intermediate double beams. When *w* is 500 μm, the proposed sensor achieves a better consistence of sensitivities along the three axes, with sensitivities of 0.302 mV/g, 0.235 mV/g and 0.347 mV/g, respectively.

**Figure 9:**
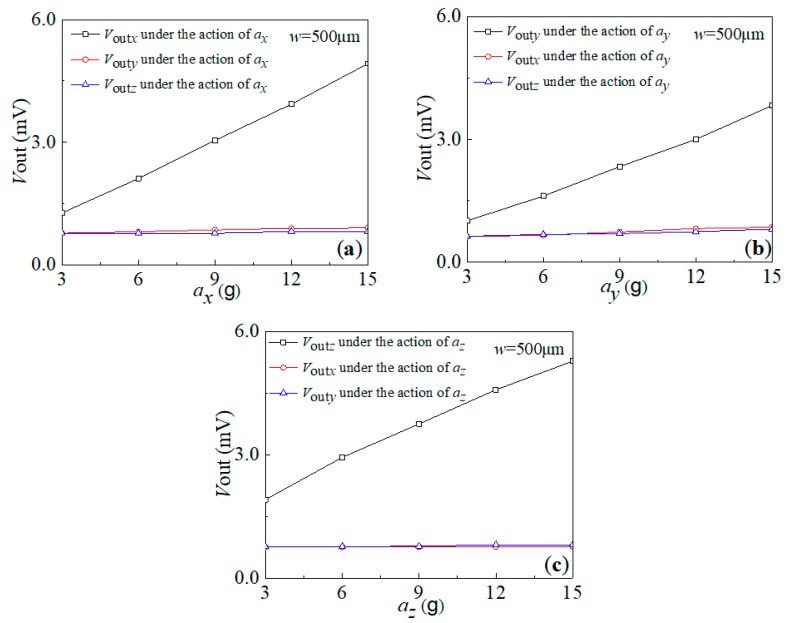
The cross-interference characteristic curves of the AS-6 sensor under three-axis acceleration along: (**a**) *x*-axis; (**b**) *y*-axis; (**c**) *z*-axis.

To further study the cross-interference characteristics of the sensitivity of the AS-6 sensor, the output voltages along the three axes at constant accelerations of *a_x_*, *a_y_* and *a_z_* were investigated at the same time, respectively. Figure 9 shows the relationship curves between the output voltages and the corresponding acceleration. As shown in Figure 9a, *V*_out*x*_ along the sensitive axis linearly increases with the increasing *a_x_*, but with slight changes in *V*_out*y*_ and *V*_out*z*_ under the same acceleration, respectively. This indicates that the *V*_out*y*_ and *V*_out*z*_ of the cross-interference caused by *a_x_* are low. Similarly, the output voltage *V*_out*y*_ (or *V*_out*z*_) linearly increases with the increasing *a_y_* (or *a_z_*), with slight changes in the other output voltages under the action of *a_y_* (or *a_z_*), indicating that the *V*_out*x*_ and *V*_out*z*_ (or *V*_out*x*_ and *V*_out*y*_) of the cross-interference caused by *a_y_* (or *a_z_*) are small as well, as shown in Figure 9b,c. The test result indicates that it is possible to achieve a high output voltage along the sensitive axis and a low cross-interference along the non-sensitive axes using the AS-6 sensor.

In addition, the characteristic parameters of the AS-6 sensor are shown in Table 4. It can be seen that, when *w* is 500 μm, maximum and minimum cross-axis sensitivities can be achieved by the proposed sensor, i.e., 0.019 mV/g and 0.001 mV/g, respectively. According to the above data analysis, it is possible to improve the consistency of sensor sensitivity and reduce the cross-interference by using the proposed scheme.

## 5. Conclusions

In summary, a three-axis accelerometer with double L-shaped beams was designed and fabricated using micro-electromechanical systems (MEMS) technology in this study. When applying acceleration to the proposed sensor chip, the corresponding acceleration along the three axes could be measured according to the output voltage changes of three Wheatstone bridges based on the elastic force theory and piezoresistive effect. To investigate the characteristics of the accelerometer, the effects of the width of intermediate double beams on the characteristics were simulated to optimize the structure size. Based on that, six types of sensors with different sizes were fabricated on silicon-on-insulator (SOI) wafers using MEMS technology and were packaged on printed circuit boards (PCB) using an electrostatic bonding process and inner lead bonding technology. The test results indicate that, at room temperature and *V*_DD_ = 5.0 V, better sensitivities along the three axes can be achieved by the resulting sensor with an optimized size, i.e., 0.302 mV/g, 0.235 mV/g and 0.347 mV/g, respectively. This study provides an effective fabrication method to further improve the sensitivity characteristics of three-axis accelerometers. 

## Figures and Tables

**Table 1:** The parameters of the six chips with different *w* values.

Type	AS-1	AS-2	AS-3	AS-4	AS-5	AS-6
*w* (μm)	250	300	350	400	450	500

**Table 2:** The resonance frequencies of the proposed sensor with different intermediate double beam widths.

Type	*w* (μm)	Resonance Frequency along *x*-axis (Hz)	Resonance Frequency along *y*-axis (Hz)	Resonance Frequency along *z*-axis (Hz)
AS-1	250	9106	8956	3211
AS-2	300	9374	7963	3221
AS-3	350	7787	8957	3314
AS-4	400	8692	8694	3250
AS-5	450	7996	8348	3109
AS-6	500	8898	8395	3270

**Table 3:** The sensitivities of the proposed sensor with different widths of intermediate double beams.

Type	*w* (μm)	*S_xx_* (mV/g)	*S_yy_* (mV/g)	*S_zz_* (mV/g)	Linearity (*x*-axis)	Linearity (*y*-axis)	Linearity (*z*-axis)
AS-1	250	0.211	0.324	0.646	3.3%	2.7%	9.1%
AS-2	300	0.135	0.163	0.633	7.6%	5.4%	6.8%
AS-3	350	0.158	0.117	0.515	7.1%	5.5%	7.2%
AS-4	400	0.369	0.271	0.504	3.0%	3.2%	8.3%
AS-5	450	0.239	0.142	0.470	5.3%	3.5%	10.0%
AS-6	500	0.302	0.235	0.347	4.1%	3.4%	3.4%

**Table 4:** The characteristic parameters of the AS-6 sensor.

	Characteristic Parameters	Resonant Frequency (Hz)	Bandwidth (Hz)	Sensitivity of the Sensor along the *x*-axis, *y*-axis and *z*-axis under Resonant Frequency (mV/g)		
Acceleration Sensor				*a_x_*	*a_y_*	*a_z_*
Sensor along *x* axis		8898	100–8000	0.302	0.011	0.004
Sensor along *y* axis		8395	100–7500	0.019	0.235	0.014
Sensor along *z* axis		3270	100–3000	0.001	0.004	0.347
